# A Robust Method: Arbitrary Shape Text Detection Combining Semantic and Position Information

**DOI:** 10.3390/s22249982

**Published:** 2022-12-18

**Authors:** Zhenchao Wang, Wushour Silamu, Yuze Li, Miaomiao Xu

**Affiliations:** Xinjiang Multilingual Information Technology Laboratory, Xinjiang Multilingual Information Technology Research Center, College of Information Science and Engineering, Xinjiang University, Urumqi 830017, China

**Keywords:** text detection, deep learning, probability map, semantic information, positional encoding

## Abstract

There is a growing interest in scene text detection for arbitrary shapes. The effectiveness of text detection has also evolved from horizontal text detection to the ability to perform text detection in multiple directions and arbitrary shapes. However, scene text detection is still a challenging task due to significant differences in size and aspect ratio and diversity in shape, as well as orientation, coarse annotations, and other factors. Regression-based methods are inspired by object detection and have limitations in fitting the edges of arbitrarily shaped text due to the characteristics of their methods. Segmentation-based methods, on the other hand, perform prediction at the pixel level and thus can fit arbitrarily shaped text better. However, the inaccuracy of image text annotations and the distribution characteristics of text pixels, which contain a large number of background pixels and misclassified pixels, degrades the performance of segmentation-based text detection methods to some extent. Usually, considering whether a pixel belongs to a text region is highly dependent on the strength of the semantic information it has and the position of the pixel in the text area. Based on the above two points, we propose an innovative and robust method for scene text detection combining position and semantic information. First, we add position information to the images using a position encoding module (PosEM) to help the model learn the implicit feature relationships associated with the position. Second, we use the semantic enhancement module (SEM) to enhance the model’s focus on the semantic information in the image during feature extraction. Then, to minimize the effect of noise due to inaccurate image text annotations and the distribution characteristics of text pixels, we convert the detection results into a probability map that can more reasonably represent the text distribution. Finally, we reconstruct and filter the text instances using a post-processing algorithm to reduce false positives. The experimental results show that our model improves significantly on the Total-Text, MSRA-TD500, and CTW1500 datasets, outperforming most previous advanced algorithms.

## 1. Introduction

With the development of information technology, the processing of text has become increasingly important. As a key part of image information processing, scene text detection technology is attracting more and more attention from researchers. The scene text detection task mainly detects the position information of text regions from natural scenes. Accurate detection results facilitate the application of technologies such as text recognition, simultaneous translation, scene analysis, image retrieval, and autonomous driving.

Traditional object detection is based on human-designed features [1,2,3] for detection, and the regions in the scene image that match these features are marked as text regions according to the designed features, thus distinguishing them from non-text regions. The detection results rely heavily on designed features, including color, gradient, texture, and other features. However, natural scene images often contain complex feature information, and the robustness of scene text detection with only one or a few artificially designed features is often not ideal. Deep learning, with its powerful feature learning capability, is widely used in machine learning research, and the features it learns tend to have a higher discriminative ability and are more robust, which makes the performance of text detection [4] greatly improved. In general, text detection can be divided into two main categories: regression-based text detection and segmentation-based text detection. Regression-based text detection is mostly inspired by traditional object detection. It treats text as a special object and detects it by estimating its bounding box and continuously regressing to approximate the ground truth. However, it has certain limitations in fitting the edges of arbitrarily shaped text due to its characteristics. Segmentation-based text detection, on the other hand, classifies the pixels of an image from the pixel level to determine if a pixel belongs to text. Then, a post-processing algorithm is used to obtain a text box, and this method can fit text with arbitrary shapes better.

Although segmentation-based methods can better fit the text shape and solve the problem of arbitrarily shaped text detection to a certain extent, there are still many challenges and problems with scene text detection for arbitrary shapes, such as (1) natural scene pictures containing rich background information, which contain a large amount of interference information similar to the text, and there are huge differences in text shape, size, and aspect ratio. How the model can better learn text-related feature information is important for accurate scene text detection. (2) Coarse text boundary annotations: the general natural scene text datasets only provide coarse-grained text boundary annotation, the text region contains a large number of background pixels, and these misclassified pixels are called ambiguous samples, which will make the model learn a large amount of misinformation and reduce the detection performance of the model to some extent. (3) Text adhesion in scene images. How to separate adjacent text becomes another task.

To address the above problems, previous research has focused on how to extract features in scene images by using n×n regular convolutional kernels. Although certain results have been achieved, the regular convolutional kernels are often prone to introduce a large amount of noise on the detection of arbitrarily shaped text in regions containing a large number of background pixels and regions with strong interference, making the detection of the model ineffective. Meanwhile, the problem of fitting text shapes and the detection of small text still suffers from a high probability of false detections as well as missed detections, although some segmentation-based techniques try to reduce the dependence on pixel prediction through orientation fields [5]. However, the performance of these methods in curved text detection is still unsatisfactory due to the unpredictability of the orientation. On adjacent text processing, PSENet [6] solves the problem of text adhesion by multi-scale text kernel and progressive expansion, and this method leads to a long post-processing time. For the problem of coarse-grained text boundary annotations, previous segmentation-based approaches often convert the segmentation results into binary maps. Since the model learning process relies heavily on the pixel classification results, these misclassified pixels often have a serious impact on the performance of the model.

We consider that position information contributes positively to the learning of the model as well as semantic information. Therefore, in this paper, we propose a segmentation-based method that uses a combination of semantic and position information. In this approach, we propose the position-encoding module (PosEM) to add position-encoding information to the image, which allows the model to better learn the implicit relationships among features, such as the distribution relationships between text pixels, textures, and colors, through this position encoding information. Meanwhile, we propose the semantic enhancement module (SEM), in which we use irregular convolution kernels that better fit the characteristics of the text, and use such convolution kernels to enable the model to extract the semantic information in the image better while reducing the noise interference caused by the regular convolution kernels. These two modules enable the model to learn feature information related to text more effectively. Then we use the position-encoding information generated by the PosEM to cluster the text pixels and solve the problem of text adhesion, and the segmentation results are converted into a probability map that can more reasonably represent the text distribution to reduce the interferences caused by coarse-grained annotations and text pixel distribution characteristics.

The main contributions of this paper are as follows:

(1) We propose an efficient position-encoding module that enhances the robustness of the model by adding position-encoding information to the image so that the model can perceive the position information during the learning process to enhance the robustness of the model, by better learning the implicit relationships of feature information in the image.

(2) We propose the semantic information enhancement module, in which we enhance the extraction of semantic information, while being able to reduce the interference caused by unnecessary noise, and fuse the features with stronger semantic information with features containing position information obtain get improved feature information.

(3) We use the position encoding information to cluster text pixels, solve the text adhesion problem, and map text instances into more reasonable probability maps, reducing the interference of ambiguous samples due to coarse-grained annotations and text distribution characteristics.

(4) To demonstrate the effectiveness of our model on arbitrarily shaped text detection, we conducted experiments on several publicly available datasets. The experimental results show that our model is highly competitive and in the advanced ranks compared to previous methods.

## 2. Related Work

As was mentioned above, traditional scene text detection relies on manually designed features, so it does not perform well in scene text detection with complex feature information. With the rise of deep learning, its powerful learning ability can learn features of text well, which improves the effectiveness of text detection to a large extent. However, scene text detection still faces the problem of large differences in the size, aspect ratio, orientation, and shape of the text. Current research is mainly based on convolutional neural networks (CNNs) for text detection. First, it uses CNNs for feature extraction and fusion of the resulting multi-scale feature maps to compensate for the limitations imposed by a single feature map. Then, by calculating the confidence level and determining whether the pixel belongs to the text. Finally, text instances are obtained by post-processing algorithms. Current scene text detection methods are divided into two main categories: regression-based text detection and segmentation-based text detection.

The regression-based method for scene text detection is based on object detection algorithms such as faster R-CNN [7], SSD [8], and mask R-CNN [9], which consider the text as a special kind of object. The text instances are predicted by continuously regressing on the bounding boxes. CTPN [10] is improved based on Faster R-CNN by generating multiple vertical anchor boxes of fixed width and then combining the anchor boxes belonging to the same text instances into the final text box. The method is effective in detecting horizontally distributed text in natural scenes but is not effective in detecting non-horizontally distributed text. RRPN [11] proposes a text detection method with a rotation angle by adding angle information to a region proposal network (RPN) and then mapping the RROI suggestion box to the feature map. The result is sent to the classifier for processing. the angle information of RRPN makes it well-suited for the detection of inclined text. EAST [12] proposes an efficient end-to-end detection method, which achieves text detection in arbitrary directions by performing offset regression on the boundaries and vertices of the text. MOST [13] uses deformable convolution to adjust the receptive field of the feature layer and proposes the Instance-wise IoU loss function. It solves the problem of insufficient receptive fields in detecting long text and poor detection of small-scale text. SegLink [14] cuts each text instance into more easily detectable directed text segments. Then it determines whether they belong to the same text instance based on the connection information between text segments. To a certain extent, it solves the problem of detecting long texts and multi-directional texts. For long text and curved text problems, LOMO [15] proposes iterative modules and shape expression modules to obtain more accurate text region locations. ContourNet [16] is designed with an adaptive RPN that effectively handles the problem of large-scale variation and achieves finer text region localization. Nevertheless, the regression-based approach still has some limitations in arbitrarily shaped scene text detection.

Segmentation-based scene text detection methods predict at the pixel level. Each pixel is classified to determine whether the pixel belongs to a text object and its connectivity with surrounding pixels to obtain a mask for the text. The pixels are then clustered by post-processing to reconstruct the text instances. PixelLink [17] predicts the connection for eight neighboring pixels of each pixel and determines whether they belong to the same text instance based on the prediction result. PSENet sets different scales of the kernel for text regions and gradually expands the detected text regions by using the progressive expansion method. This effectively solves the problem of separating adjacent text instances in the process of reconstructing text instances. To address the problem of the high time overhead of PSENet post-processing, PAN [18] proposed a new pixel clustering method with faster prediction speed while maintaining high precision. SPCNet [19] proposes a text context module to complement the contextual information while using a re-score mechanism to effectively suppress false positives. DBNet [20] proposed a module named differentiable binarization, which solves the problem that standard binarization cannot be optimized and learned together with the network due to its non-differentiability. This design enables the self-adaptive setting of binarization thresholds for the network, which improve the detection performance greatly.

## 3. Proposed Method

**Figure 1:**
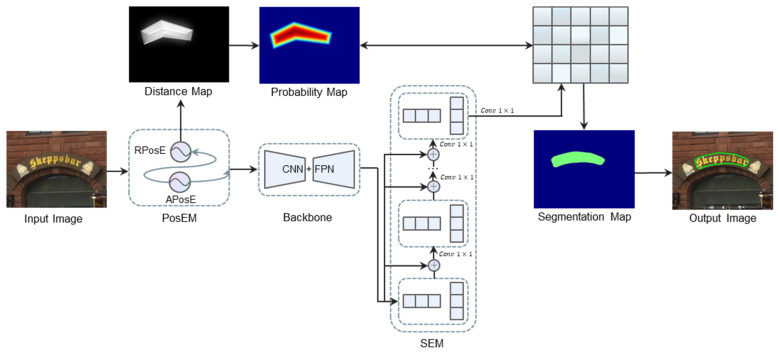
It shows the overall framework of our proposed method. ⊕ Means the concat operation.

The overall structure of our proposed model is shown in Figure 1.

We use the positional encoding module (PosEM) to get the absolute positional encoding information of the image, and then feed the absolute positional encoding together with the image to the backbone network for feature extraction, and calculate the relative position encoding. In order to make full use of the multi-layer feature information, we use the feature pyramid network [21] (FPN) to fuse the multi-scale feature layer information. The semantic enhancement module (SEM) is then used to obtain feature maps with strong semantic information and fused them to enhance the semantic information in the feature maps while using iteration to strengthen the process. Finally, the positional information generated by PosEM is used to cluster pixels to reconstruct text instances and produce probability maps that can more reasonably represent the text distribution.

### 3.1. Positional Encoding Module (PosEM)

**Figure 2:**
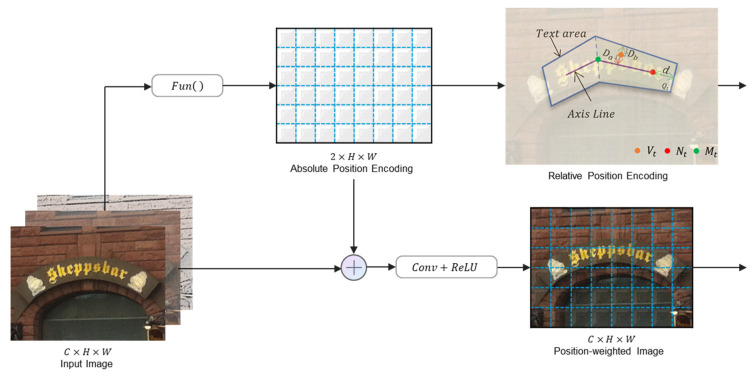
It shows the details of PosEM. The absolute position encoding module adds position information to the input image, which allows the model to perceive the position information in the process of learning text features. The relative position encoding module reduces the effect of coarse boundaries on model learning.

The structure of PosEM is shown in Figure 2.

In this part of our work, the following analysis was performed.

Compared to the fully connected network [22] (FCN), the convolutional kernel of the convolutional neural network (CNN) can be considered as a local filter that improves the efficiency of image processing through local links in a limited space. Precisely for this reason, although the convolutional kernel can perceive information about the current region, this local link is usually not considered to be able to perceive the current position where it is located.

The image contains rich and complex information, and the distribution of this information is extremely uneven. The effective information contained in each position is not the same for different tasks. The scene text detection task focuses more on learning the information and the features of the text. Through our analysis, we found that although the distribution of text regions in images is irregular, the distribution of features such as text pixels, textures, and colors in text regions is unique, for example, pixels in the center of text regions have a high probability of being text pixels, while pixels in edge regions have a low probability of being text pixels. It is because the edge region contains more background information due to inaccurate annotations of text and the text pixel distribution characteristics, and the probability that pixels in these regions are text pixels is low, but they are also segmented as text regions. These ambiguous samples will cause interference with the model learning.

Based on the above analysis, we added the PosEM to the CNN to help the model learn relationships in text features more effectively and minimize the impact of other interfering information in the image. Due to the uncertain size of the image, the approach of positional encoding is required to be extensible, so we adopt a scalable and efficient way to generate absolute positional encoding for the image. In this encoding operation, we resolve the position coordinates from two dimensions. For any point v(i,j) in the image, its position information is encoded as

(1) $$pos\left(i,j\right)=Fun_{pos}\left(v\left(i,j\right)\right)$$

where pos(i,j) denotes the generated position encoding and $Fun_{pos}\left(\;\right)$ is the position encoding function. and the position information is combined with the original image as the input of the network:

(2) $$F_{pos}=Relu\left(BN\left(Conv\left(P_{pos}\right)\right)\right)$$

where

(3) $$P_{pos}=\left(v\left(i,j\right)⊕pos\left(i,j\right)\right)$$

so that the CNN perceives position information during the process of convolution and thus learns the implied relationships in the feature distribution of the image. Such encoding can provide clear position information with little additional computational cost.

**Figure 3:**
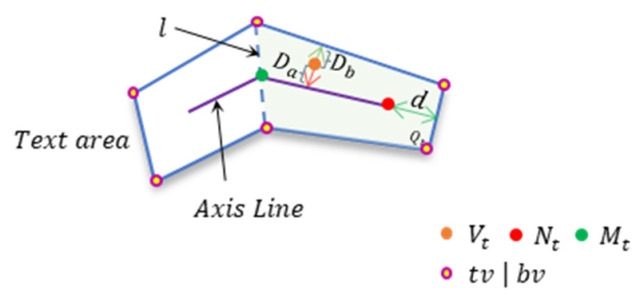
It shows the details of relative position encoding.

Moreover, to avoid the noise from inaccurate text annotation, etc., on model learning, we treat the text region as a probability map instead of a binary map. To generate the corresponding probability maps, we construct relative position encoding for arbitrarily shaped text. The construction method is as follows: we first construct the axis line by dividing the boundary points of the long edge of the text area into two point sets $V_{1}=\left\{tv_{1},tv_{2},\cdots ,tv_{n}\right\}$ and $V_{2}=\left\{bv_{1},bv_{2},\cdots ,bv_{n}\right\}$, and generate the positioning connection line $L=\left\{l_{1}=\left(tv_{1},bv_{1}\right),l_{2}=\left(tv_{2},bv_{2}\right),\cdots ,l_{n}=\left(tv_{n},bv_{n}\right)\right\}$, determine the axis line positioning points according to the edge points and the positioning connection line, and connect the positioning points to form the axis line. To make the generated position encoding more reasonable, we scaled the axis lines with a shrinkage scale as follows:

(4) $$d=\frac{S\left(1-r^{2}\right)}{C}$$

where S is the area of the polygon region, C is the perimeter of the polygon region, r is the shrinkage ratio, which is empirically set to 0.4, and shrink the axis line inward at both ends by d. Then we divide the text region into multiple quadrilateral $Quad=\left\{Q_{1},Q_{2},\cdots ,Q_{n-1}\right\}$ and calculate the distances $D_{a}$ and $D_{b}$ of the pixels in the quadrilateral $Q_{t},\;\;t\in \left[1,n-1\right]$ relative to the axis and the boundary, respectively, using absolute position encoding, where the belonging of the pixels is defined by the quadrilateral, and since they are relative distances, we call this distance information relative position information (as is shown in Figure 3).

(5) $$r=\frac{\vec{M_{t}V_{t}}\left(i,j\right)\cdot \vec{M_{t}N_{t}}}{{\left|\vec{M_{t}N_{t}}\right|}^{2}}$$

(6) $$D_{a}=D_{b}=\{\begin{array}{ll} |\vec{M_{t}V_{t}}|, & r\leq 0\\ |\vec{N_{t}V_{t}}|, & r\geq 1\\ |\vec{{V^{\prime }}_{t}V_{t}}|, & others \end{array}$$

where $M_{t}$, $N_{t}$ are the two endpoints of the axis of $Q_{t}$,$\;\;V_{t}\left(i,j\right)\in Q_{t}$, and ${V^{\prime }}_{t}$ is the foot point of $V_{t}$ on MN.

### 3.2. Semantic Enhancement Module (SEM)

**Figure 4:**
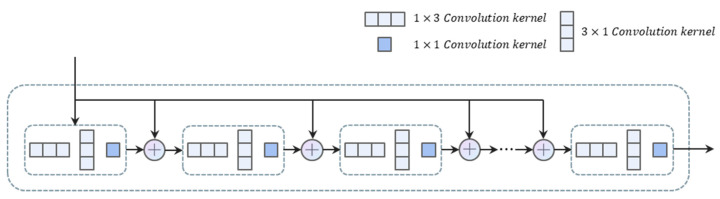
It shows the details of SEM.

In the segmentation-based scene text detection method, the performance of segmentation can be significantly improved with as comprehensive and accurate semantic information as possible. Therefore, in this module, we focus on how to obtain more accurate semantic information. The architecture of the SEM is shown in Figure 4. We use irregular [23] convolutional kernels, feature information fusion, and iterative methods [24] to obtain more semantic information. This process is strengthened to obtain more complete feature information.

First, we used non-standard 1×3 and 3×1 convolution kernels. Because we note that different from instance objects in general object detection, text instances usually have a large aspect ratio, this feature makes the standard n×n convolution kernel limited in scene text detection. This phenomenon arises because the square convolutional kernel expands the receptive field while introducing a large amount of noise, which has an impact on the learning effect of the model. If we want to reduce the effect of the noise introduced by the square convolutional kernel, the size of the receptive field will be limited, which imposes a limitation on the model for the detection of long texts. The non-standard 1×3 convolution kernel is more compatible with the characteristics of text instances in terms of shape and size, so we use this non-standard convolution kernel for feature extraction of text instances. This convolutional kernel has several advantages. On the one hand, the features extracted by this convolutional kernel have stronger semantic information. On the other hand, it also avoids the effect of the noise introduced by the standard square convolutional kernel to expand the receptive field, and it also reduces the learning parameters of the model.

Next, to further fuse the semantic and position information to obtain more complete features, we superimposed the feature map with strong semantic information with the feature map with implicit position information.

(7) $$F_{1}=Relu\left(Conv_{1\times 3}\left(F_{0}\right)\right)$$

(8) $$F_{i}=Conv_{1\times 3}\left(Relu\left(Conv_{1\times 1}\left(F_{0}⊕F_{i-1}\right)\right)\right)$$

(9) $$F_{out}=Sigmoid\left(F_{n}\right)$$

The feature extraction process was subsequently enhanced using an iterative approach.

### 3.3. Post-filtering Algorithm

To solve text adhesion and reduce the interference caused by inaccurate annotations of text boundaries and distribution characteristics of text pixels, we reconstruct text instances by clustering text pixels based on relative position encoding and use a probability value conversion function,

(10) $$LF=\left(1-\alpha \right)\left(\frac{-D_{a}}{D_{a}+D_{b}}\right)+1$$

where α set to 0.01 to convert the binary map into a more reasonable probability map representing text probabilities.

Considering that the threshold filtering algorithm under a single condition has large limitations, and is prone to misclassify text instances with strong background interference, we propose to use both the average confidence and area of the predicted text instance regions to design the post-filtering algorithm. This filtering algorithm integrates two plausible conditions for text instances and can effectively avoid misclassification in the case of a single condition.

### 3.4. Loss Function

Our loss function is expressed as the sum of the losses of multiple quadrilaterals in a text instance.

(11) $$L=\sum_{i=1}^{n}\lambda_{i}\cdot loss\left(Q_{i}\right)$$

where $\lambda_{i}$ is the weight of the loss in the corresponding quadrilateral region and the sum of all λ is equal to 1.

(12) $$\lambda_{i}=\frac{S\left(Q_{i}\right)}{\sum_{j=1}^{n}S\left(Q_{j}\right)}$$

The loss in each quadrilateral region is the Mean Square Error (MSE) of all pixels in the quadrilateral,

(13) $$loss_{Q_{i}}=\frac{1}{Q_{i}}\underset{v\epsilon Q_{i}}{\sum }{\left(\left\|G_{Q_{i}}\left(v\right)-P_{Q_{i}}\left(v\right)\right\|\right)}^{2}$$

where $G_{Q_{i}}$ is the corresponding quadrilateral in the ground truth and $P_{Q_{i}}$ is the quadrilateral in the prediction graph, and v represents the pixels in $Q_{i}$. To overcome the problem of unbalanced positive and negative samples, we adopt an online hard example mining [25] (OHEM) strategy for loss.

## 4. Experiments

In this section, we summarize the public datasets used for the experiments and our experimental configurations. We conducted relevant ablation experiments on these public datasets and we compared the effectiveness with other detection algorithms through comparison experiments. Three main performance parameters, precision (P), recall (R), and F-measure (F), are considered to evaluate the detection performance of the model.

### 4.1. Datasets

To fully validate the performance of our model, we validate our model on the following challenging public datasets.

Total-Text [26] is a publicly available dataset consisting of 1555 scene images, with a train set containing 1255 images and a test set containing 300 images. This dataset uses polygonal annotation boxes because these scene images contain much multi-directional text and curved text. The images also contain a large amount of background noise that is similar to the text regions.

The CTW1500 [27] dataset consists of 1500 scene images, of which the train set consists of 1000 scene images and the rest 500 are the test set. Similarly, it contains a large number of arbitrarily shaped text in the images, and the text regions are marked with polygonal bounding boxes.

The MSRA-TD500 [28] dataset contains 500 scene images, in which the text includes Chinese and English. The train set is 300 images randomly selected from the original dataset, and the remaining 200 images constitute the test set. The diversity of their text sizes and text spacing, as well as having a similar background to the text, make this dataset a challenge.

### 4.2. Implementation Details

The experiments in this paper were conducted in a Linux environment, using Python 3.7 as the programming language, Pytorch 1.2 as the implementation framework, CPU: Intel(R) Xeon(R) CPU E5-2640 v4 @ 2.40GHz; GPU: Tesla V100 16G. All the following experiments were conducted on a single GPU.

We used ResNet-50 [29] which pre-trained on the ImageNet dataset, as our backbone and the rest as described in the paper. The network uses stochastic gradient descent [30] (SGD) as the optimizer, with the initial learning rate set to 0.01 and the current learning rate dynamically adjusted to the initial learning rate multiplied by $0.9{\;}^{\left[epoch/100\right]}$ where epoch is the current number of iterations. Our model is simply pre-trained on the SynthText [31] dataset, and all datasets are officially provided. We resized the image size to 800 × 800 and set the batch to 4 for 800 epochs on the Total-Text dataset. On the CTW1500 dataset, we resized the image size to 640 × 640 and set the batch to 6, and conducted 800 epochs of training. On the MSRA-TD500 dataset, we resized the images to 640 × 640 and set the batch to 6 for 900 epochs. To improve the robustness of the model, we performed data augmentation on the training data by random rotation, random cropping, and random flipping.

### 4.3. Ablation Experiments

We conducted ablation experiments on the publicly available datasets MSRA-TD500 dataset and Total-Text dataset to verify the effectiveness of our proposed PosEM and SEM. First, we verified the experimental effect of the baseline without including PosEM and SEM, then we verified the effect of adding only PosEM based on the baseline, and adding only SEM based on the baseline, respectively. Finally, we validated the complete model with the addition of the PosEM and SEM. Note that the “baseline” mentioned in our experiments refers to the model reproduced from the PSENet open-source repository. The results are shown in Table 1, where the image sizes are resized to 800 and 640, respectively.

From Table 1, we can see that our proposed PosEM can effectively improve the performance of Resnet-50. PosEM improves precision, recall, and F-measure by 1.46%, 2.92%, and 1.56%, respectively, on the Total-Text dataset. PosEM improved precision, recall, and F-measure by 3.73%, 2.95%, and 1.56%, respectively, on the MSRA-TD500 dataset. This is because PosEM can help the model to better learn the implicit relationship of feature information, which helps the model to better identify text regions. At the same time, the location information is used to supervise the learning of the model, which reduces the interference of noise in the text region on the model learning.

As shown in Table 1, the SEM module can help the model to better extract the semantic information of the text area, which effectively enhances the model’s ability to discriminate between text and non-text areas. Therefore, SEM also brings a certain gain effect to the performance of the model. On the Total-Text dataset, the accuracy and F-measure are improved by 1.73% and 1.15%, respectively. On the MSRA-TD500 dataset, the accuracy and F-measure are improved by 4.53%and 1.81%respectively.

Overall, the precision, recall, and F-measure of the baseline algorithm on the Total-Text dataset are 86.28%, 80.44%, and 83.25%, respectively, while our method improves 3.01%, 2.95%, and 2.99% in the three metrics to 89.29%. 83.39% and 86.24%. On the MSRA-TD500 dataset, the precision, recall, and F-measure of the baseline algorithm are 83.25%, 80.93%, and 82.07%, respectively, while our method improves the three metrics by 6.28%, 2.39%, and 4.24%, reaching 89.53%, 83.32%, and 86.31%. It can be seen that our performance is significantly better than the baseline algorithm.

### 4.4. Comparison Experiments

In this section, to fully validate the performance of our model, we compare it with previous methods on several publicly available datasets including Total-Text, MSRA-TD500, and CTW1500, as shown in Table 2, Table 3 and Table 4. We keep the aspect ratio of images and resize them to the appropriate size for testing.

As seen in Table 2, on the Total-Text dataset, our algorithm improves 5.6%, 5.48%, and 5.55% in precision, recall, and F-measure, respectively, compared to PSENet, a segmentation algorithm that also uses ResNet-50 as its backbone network. In terms of precision and F-measure, our algorithm outperforms other algorithms by at least 0.32% and 0.42%. This is due to our algorithm’s ability to fit text boundaries better and its ability to discriminate textual regions from non-textual regions. Although we have some advantages in precision and F-measure, our algorithm recall is 1.49% lower than the best performance. This is caused by the large character spacing of the text.

As seen in Table 3, on the MSRA-TD500 dataset, the F-measure of our algorithm is 86.73%, which is only 0.47% lower than the DBNet++ algorithm with the best performance. Compared to other algorithms such as PAN, DBNet, and DRRG, our F-measure is at least 1.65% higher. This shows that our algorithm is still competitive in multilingual and multi-directional text detection.

As seen in Table 4, the accuracy of our algorithm is 86.91% on the CTW1500 dataset, which is only 0.99% lower than DBNet++, which has the best performance. Our F-measure value of 83.59% is generally consistent with the TextMountain, ContourNet, and DBNet algorithms. It is 1.81% lower in F-measure compared to the best performance. This is because CTW1500 contains many texts with large character spacing, which makes the algorithm incomplete in fitting the text edges due to the limited receptive field. Nevertheless, our algorithm still outperforms PSENet, a segmentation algorithm that also uses ResNet-50 as its backbone network, in terms of overall performance.

As shown in Table 2, Table 3 and Table 4, in terms of the inference speed of the model, the FPS of our model is greater than 25, which means that our algorithm is nearly real-time .

### 4.5. Visualization Results

**Figure 5:**
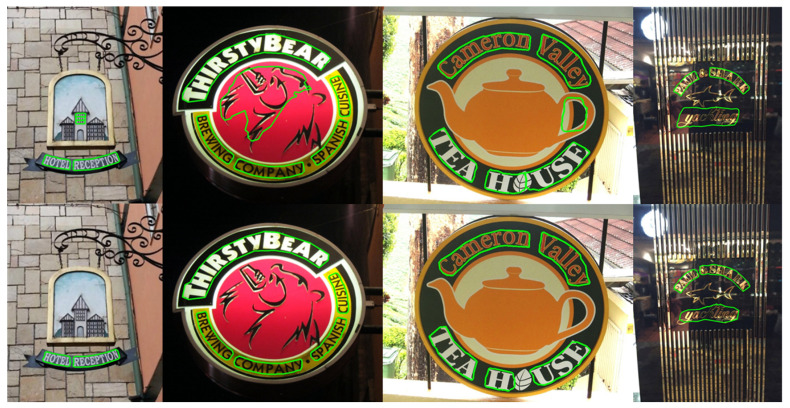
The visualization compares the detection results of our method with other methods when performing text detection in regions similar to text and regions with strong interference. The upper half is the detection results of other methods, which often detect non-text regions as text regions by mistake. The lower half is the detection result of our model, which shows that our model can distinguish text and non-text regions well in this case.

**Figure 6:**
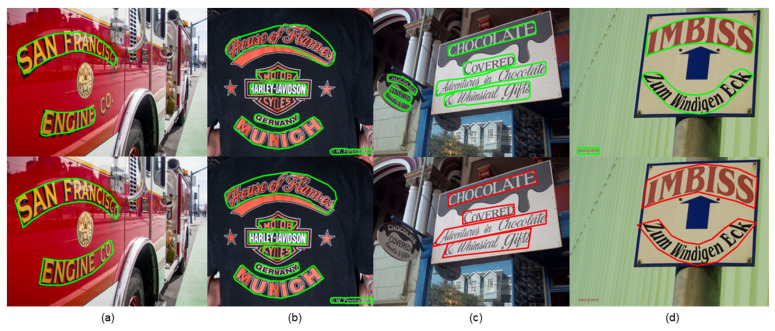
The visualization results (**a**,**b**) show the effectiveness of our model in detecting adjacent text and text with large-scale differences. Our model can separate adjacent text well during detection and detects text with large-scale differences well. (**c**,**d**) show that even if only coarse text annotations are provided, our model can reduce the impact of coarse annotations on learning text features, and the boundaries in the detection results fit the text edges well.

**Figure 7:**
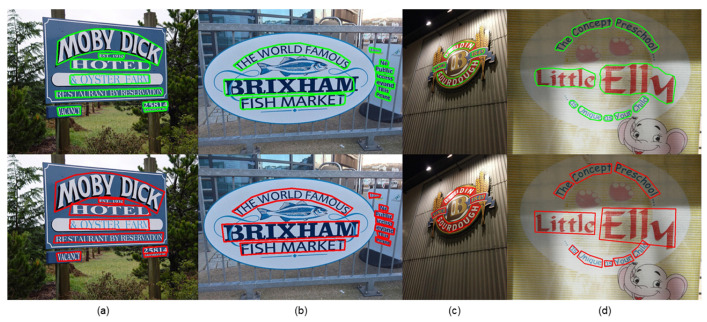
Visualization results (**a**,**b**) show that our method works better in the lower half compared to other methods in the upper half in detecting text and art words with different viewpoints. (**c**,**d**) further show the detection of text ignored during text annotation, where the upper half is the detection effect of our model and the lower half is the text annotation ground truth.

We visualize the detection results and compare them with those of other methods, as shown in Figure 5, Figure 6 and Figure 7.

We visualize the detection results and can see that our method can fit the edges of text shapes well in the detection of arbitrarily shaped text. We further validate the superiority of our model by comparing the detection results with those of other methods, as shown in Figure 5, Figure 6 and Figure 7. Figure 5 demonstrates that our method detects better in regions similar to the text and regions with strong interference. Figure 6 demonstrates that our method works well on the problems of adjacent text separation and large-scale difference text detection. Figure 7a,b show that our model also works well for abstract word and multi-angle shot text detection. Figure 7c,d demonstrates that our model can correctly detect even text that is overlooked during text annotation. It can be seen that our model still has strong robustness in complex situations.

## 5. Conclusions

In this paper, we propose a new text detection method for scenes with arbitrarily shaped text.

We use the PosEM to add both the absolute position information and the relative position information to the images to help the model learn the text features, enabling the model to better learn the implicit connections between the features. Then we use the SEM to enhance the semantic information of the extracted features so that the model can further learn the semantic information in the text instances and fuse the feature information to obtain better feature information. Finally, the post-processing algorithm is used to reconstruct the text instances by clustering the pixels based on the position information and filtering the detection results to reduce false positives. We validate our method on the publicly available datasets Total-Text, MSRA-TD500, and CTW1500, and the results show that the algorithm in this paper can achieve good results for arbitrary shape text detection.

We will explore how to improve the performance of the model in the following aspects, (1) how to solve the problem of not being able to cover large character-spacing text instances well due to the limited receptive field. (2) Further improving the inference speed of the model. (3) Applying our algorithm to text recognition and developing an end-to-end scene text recognition model.

## Figures and Tables

**Table 1:** Results of the ablation experiments on the Total-Text and MSRA-TD500 datasets.

Backbone	PosEM	SEM	Total-Text			MSRA-TD500		
			P (%)	R (%)	F (%)	P (%)	R (%)	F (%)
ResNet-50	✕	✕	86.28	80.44	83.25	83.25	80.93	82.07
ResNet-50	√	✕	87.74	83.36	85.49	86.98	83.88	83.63
ResNet-50	✕	√	88.01	81.06	84.4	87.78	80.32	83.88
ResNet-50	√	√	89.29	83.39	86.24	89.53	83.32	86.31

**Table 2:** Detection results on the Total-Text dataset. “∗” indicates using deformable-ResNet-50 as backbone and “†” indicates using external MLT17 data for pre-training. The best results of all measures are bold.

Method	P (%)	R (%)	F (%)	FPS
TextSnake [32]	82.7	74.5	78.4	-
ATTR [33]	80.9	76.2	78.5	10.0
MSR [34]	85.2	73.0	78.6	-
TextDragon [35]	85.6	75.7	80.3	-
TextField [5]	81.2	79.9	80.6	-
PSENet-1s † [6]	84.02	77.96	80.87	3.9
LOMO [15]	87.6	79.3	83.3	-
CRAFT † [36]	87.6	79.9	83.6	-
DBNet * [20]	87.1	82.5	84.7	32.0
ContourNet [16]	86.9	83.9	85.4	3.8
PAN [18]	89.3	81.0	85.0	**39.6**
DRRG † [37]	86.54	**84.93**	85.73	-
TextFuseNet [38]	87.5	83.2	85.3	7.1
Boundary [39]	85.2	83.5	84.3	-
DBNet++ * [40]	88.9	83.2	86.0	28
**Ours**	**89.62**	83.44	**86.42**	26.2

**Table 3:** Detection results on the MSRA-TD500 dataset. “*” indicates using deformable-ResNet-50 as backbone and “†” indicates using external MLT17 data for pre-training. The best performance is given in bold.

Method	P (%)	R (%)	F (%)	FPS
SegLink [14]	86.0	70.0	77.0	8.9
TextSnake [32]	83.2	73.9	78.3	1.1
ATTR [33]	85.2	82.1	83.6	-
MSR [34]	87.4	76.7	81.7	-
TextField [5]	87.4	75.9	81.3	5.2
CRAFT † [36]	88.2	78.2	82.9	8.6
DBNet * [20]	**91.5**	79.2	84.9	**32.0**
PAN [18]	84.4	**83.8**	84.1	30.2
DRRG † [37]	88.05	82.30	85.08	-
DBNet++ * [40]	**91.5**	83.3	**87.2**	29
**Ours**	90.1	83.6	86.73	29.5

**Table 4:** Detection results on the CTW1500 dataset. “*” indicates using deformable-ResNet-50 as backbone and “†” indicates using external MLT17 data for pre-training. The best performance is given in bold.

Method	P (%)	R (%)	F (%)	FPS
TextSnake [32]	67.9	**85.3**	75.6	-
MSR [34]	84.1	79.0	81.5	-
TextDragon [35]	84.5	82.8	83.6	-
TextField [5]	83.0	79.8	81.4	-
PSENet-1s † [6]	84.8	79.7	82.2	3.9
LOMO [15]	85.7	76.5	80.8	-
CRAFT † [36]	86.0	81.1	83.5	-
DBNet * [20]	86.9	80.2	83.4	22.0
ContourNet [16]	83.7	84.1	83.9	4.5
PAN [18]	86.4	81.2	83.7	39.8
DRRG † [37]	85.93	83.02	84.45	-
TextFuseNet [38]	85.8	85.0	**85.4**	**7.3**
TextMountain [41]	82.9	83.4	83.2	-
DBNet++ * [40]	**87.9**	82.8	85.3	26
**Ours**	86.91	80.52	83.59	25.1

## Data Availability

Not applicable.
